# RNA-seq reproducibility of *Pseudomonas aeruginosa* in laboratory models of cystic fibrosis

**DOI:** 10.1128/spectrum.01513-24

**Published:** 2024-12-03

**Authors:** Rebecca P. Duncan, Gina R. Lewin, Daniel M. Cornforth, Frances L. Diggle, Ananya Kapur, Dina A. Moustafa, Yasmin Hilliam, Jennifer M. Bomberger, Marvin Whiteley, Joanna B. Goldberg

**Affiliations:** 1 Division of Pulmonary, Asthma, Cystic Fibrosis, and Sleep, Department of Pediatrics, Emory University School of Medicine, Atlanta, Georgia, USA; 2 Emory-Children’s Cystic Fibrosis Center, Atlanta, Georgia, USA; 3 Center for Microbial Dynamics and Infection, School of Biological Sciences, Georgia Institute of Technology, Atlanta, Georgia, USA; 4 Department of Microbiology and Molecular Genetics, University of Pittsburgh, Pittsburgh, Pennsylvania, USA; University of Dundee, Dundee, United Kingdom

**Keywords:** RNA-seq, *Pseudomonas aeruginosa*, reproducibility, epithelial cell model, SCFM2, cystic fibrosis

## Abstract

Reproducibility is a fundamental expectation in science and enables investigators to have confidence in their research findings and the ability to compare data from disparate sources, but evaluating reproducibility can be elusive. For example, generating RNA sequencing (RNA-seq) data includes multiple steps where variance can be introduced. Thus, it is unclear if RNA-seq data from different sources can be validly compared. While most studies on RNA-seq reproducibility focus on eukaryotes, we evaluate bias in bacteria using *Pseudomonas aeruginosa* gene expression data from five laboratory models of cystic fibrosis. We leverage a large data set that includes samples prepared in three different laboratories and paired data sets where the same sample was sequenced using at least two different sequencing pipelines. We report here that expression data are highly reproducible across laboratories. In addition, while samples sequenced with different sequencing pipelines showed significantly more variance in expression profiles than between labs, gene expression was still highly reproducible between sequencing pipelines. Further investigation of expression differences between two sequencing pipelines revealed that library preparation methods were the largest source of error, though analyses to identify the source of this variance were inconclusive. Consistent with the reproducibility of expression between sequencing pipelines, we found that different pipelines detected over 80% of the same differentially expressed genes with large expression differences between conditions. Thus, bacterial RNA-seq data from different sources can be validly compared, facilitating the ability to advance understanding of bacterial behavior and physiology using the wide array of publicly available RNA-seq data sets.

**IMPORTANCE:**

RNA sequencing (RNA-seq) has revolutionized biology, but many steps in RNA-seq workflows can introduce variance, potentially compromising reproducibility. While reproducibility in RNA-seq has been thoroughly investigated in eukaryotes, less is known about pipelines and workflows that introduce variance and biases in bacterial RNA-seq data. By leveraging *Pseudomonas aeruginosa* transcriptomes in cystic fibrosis models from different laboratories and sequenced with different sequencing pipelines, we directly assess sources of bacterial RNA-seq variance. RNA-seq data were highly reproducible, with the largest variance due to sequencing pipelines, specifically library preparation. Different sequencing pipelines detected overlapping differentially expressed genes, especially those with large expression differences between conditions. This study confirms that different approaches to preparing and sequencing bacterial RNA libraries capture comparable transcriptional profiles, supporting investigators’ ability to leverage diverse RNA-seq data sets to advance their science.

## INTRODUCTION

A universally valued quality of rigorous science is reproducibility—the ability to obtain consistent results from independent experiments (1, 2). Reproducibility is important because it increases confidence in research findings and enables investigators to combine data from multiple sources. However, despite the widespread value placed on reproducibility in science, knowledge of reproducibility in specific experiments is often, paradoxically, limited. For example, most investigators and many journals are not interested in publishing confirmatory studies of previously published results. Reproducibility may be assumed based on a consistent result arising from the replication of an experiment by the same investigator under identical conditions. However, confidence in a result is enhanced when it can be confirmed in independent experiments by different investigators and/or different experimental conditions (1, 2).

Reproducibility in life science can be achieved by controlling biological variance (e.g., through using identical genotypes, cell lines, or strains), but variance can also arise from complex tools and pipelines used in biological research. One widely used tool in biology is RNA sequencing (RNA-seq). RNA-seq has revolutionized basic research by enabling biologists to quantify global transcriptional responses of any organism to different conditions or environments. However, RNA-seq technology is also complex, requiring multiple steps that can introduce variance and bias (3); this could potentially compromise the ability to reliably reproduce findings in different studies. Community and individual efforts have identified several sources of variance in RNA-seq data, including sequencing facility, sequencing platform, read depth, normalization approaches, reference annotation, data analysis pipeline, and library preparation protocols (4–8). While these prior studies offer a valuable assessment of reproducibility and sources of variance in RNA-seq data, they have generally focused on commercially available reference RNA from human cell lines rather than empirical sequence data from replicate samples generated in multiple independent laboratories. Thus, these studies fail to capture the impact of variance introduced by different investigators and different laboratory environments. Furthermore, while these previous studies assess variance arising from library preparation methods, they focus on approaches for enriching mRNA in eukaryotes (poly-A enrichment vs ribo-depletion). It is unclear how differences in library preparation might affect variance within other systems. For example, in bacterial RNA-seq data sets, the large number of ribo-depletion approaches, library preparation kits, and sequencing facilities could contribute to variance. Understanding sources of variance, including different laboratory environments and sequencing pipelines, in RNA-seq data will thus inform future design and interpretation of transcriptional profiles.

Here, we examine RNA-seq reproducibility using *Pseudomonas aeruginosa* in laboratory models developed to study infection in the lungs of people with the genetic disease, cystic fibrosis (CF). CF is caused by a defective cystic fibrosis transmembrane regulator (CFTR) protein, a chloride and bicarbonate channel that facilitates mucus clearing from airway surfaces. Mucus (sputum) buildup in airways leads to chronic bacterial infections with *P. aeruginosa* and other pathogens (9, 10), which, coupled with the patient’s inflammatory immune response, compromises lung function and is the leading cause of morbidity in CF patients (10). A number of laboratory models have been developed to elucidate *P. aeruginosa* infection dynamics in CF, including a defined synthetic CF sputum medium (SCFM2) (11, 12) and human bronchial epithelial cell systems (13–17) that accurately capture *P. aeruginosa* transcriptional profiles in CF sputum (17, 18). In this study, we leverage RNA-seq datasets generated in three labs and sequenced with five different pipelines to answer two overarching questions: first, how does generating data at different times, in different labs, and with different sequencing pipelines influence variance in RNA-seq data? Second, does this variance impact the reproducibility of results that employ common ways to analyze data, such as differential expression analyses? We show that overall, gene expression is highly reproducible, with slightly higher variance in the expression of samples with alternate library preparation approaches. Importantly, different sequencing pipelines still detected 22 of the same differentially expressed genes with high fold changes between conditions in at least one of the pipelines. This work demonstrates that RNA-seq data are comparable between labs and sequencing pipelines, offering insights to anyone seeking answers to biological questions through transcriptomics.

## RESULTS AND DISCUSSION

### Gene expression is reproducible between laboratories and sequencing pipelines

**Fig 1:**
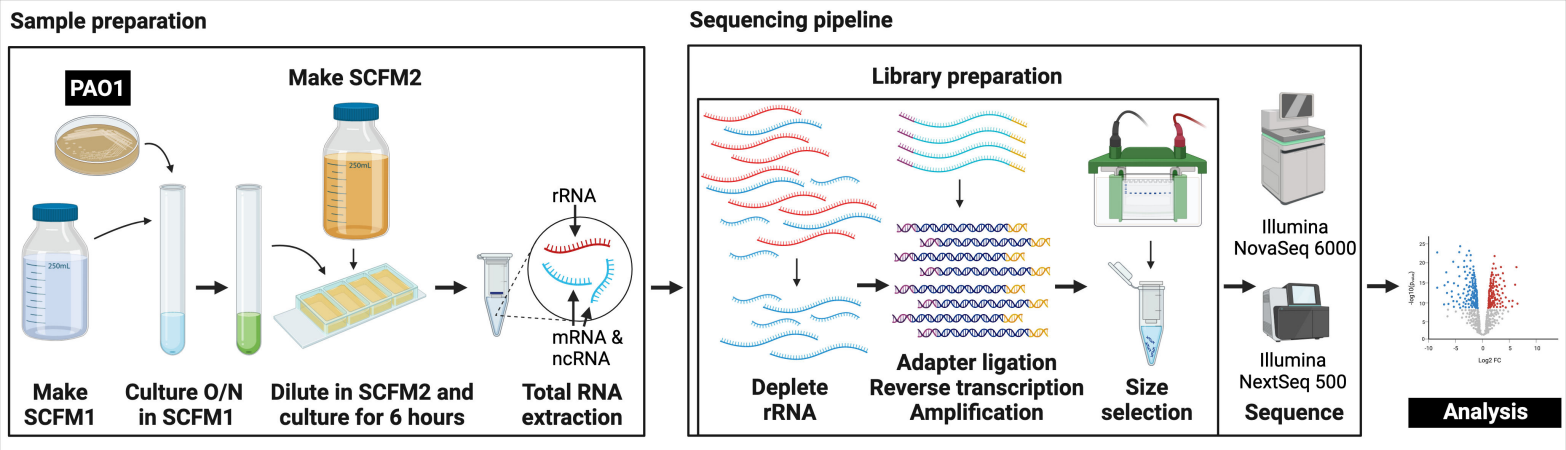
Steps that can introduce variance in the steps for producing RNA-seq data from PAO1 grown in SCFM2. Except for the PAO1 strain used and the data analysis step (white text in black boxes), which were the same for all samples and datasets reported here, all steps can introduce variance into RNA-seq data. Variance in sample preparation can be introduced by different investigators, media batches, days when samples are prepared, or laboratories. Downstream variance can be introduced by ribosomal RNA (rRNA) depletion and library preparation kits, and/or sequencing platforms used. In this study, we were able to distinguish between sample preparation, which collectively includes culture preparation, culturing, and RNA extraction, and sequencing pipeline, which collectively includes rRNA depletion, cDNA library preparation, size selection, and sequencing. The sequencing pipeline shown here is based on sequencing pipeline A (see Table 2 and Table S1). O/N, overnight. This figure was created in BioRender.

The goal of this work was to understand sources of variance in bacterial RNA-seq data. To accomplish this, we sequenced the transcriptomes of *P. aeruginosa* grown in five different laboratory models of CF (Table 1). Models included an *in vitro* synthetic sputum medium (SCFM2) that mimics the CF lung environment and multiple airway epithelial cell models, which are commonly used to study CF chronic respiratory infections (11–16, 19, 20). The sample preparation workflow for SCFM2 samples is outlined in Fig. 1 and includes culture preparation, culturing, and RNA extraction. Importantly, labeled steps represent places where variance could be introduced. SCFM2 samples were prepared across our three laboratories, and airway epithelial cell models were prepared in the lab of JMB. Both SCFM2-grown *P. aeruginosa* and airway epithelial cell models were sequenced with at least one of five sequencing pipelines, where “sequencing pipeline” refers to the steps of library preparation and sequencing (Fig. 1).

**TABLE 1:** Sample types and sequencing pipelines used^*a*^

Sample types	Total number of samples (sequencing runs)	Sequencing pipelines^*b*^	Source laboratory
SCFM2	15 (27)	A B^*c*^ C D E	Whiteley Goldberg Bomberger
Airway epithelial cell models			
AEC	3 (6)	A B	Bomberger
epiSCFM2	3 (6)	A B	Bomberger
epiSCFM2 (epithelial cells)	6 (9)	A B D	Bomberger
epiSCFM2 (apical space)	6 (9)	A B D	Bomberger

^*a*^ *Pseudomonas aeruginosa* PAO1 was cultured in all models.

^*b*^ See Table 2 and Dataset S1 for details on each pipeline for each sample.

^*c*^ Some SCFM2 samples sequenced with pipeline B were not sequenced with other pipelines.

**TABLE 2:** SCFM2 sequencing pipeline steps^*a*^

Sequencing pipeline ID	rRNA depletion kit	rRNA depletion mechanism	Library prep kit	RT primer annealing target	Sequencing platform
A^*b*^	microbExpress	Oligo capture and magnetic bead separation	NEBNext sRNA Library Prep Set for Illumina	Adapters	Illumina NextSeq 500 Illumina NovaSeq 6000
B	N/A (integrated with library preparation)	Proprietary enzymatic depletion	Zymo-Seq RiboFree Total RNA Library Prep Kit	Random hexamers	Illumina NovaSeq 6000
C	QIAseq FastSelect 5S/16S/23S	Nucleic acid binding to block reverse transcription	QIASeq Total Stranded RNA Library Prep Kit	Random hexamers	Illumina NextSeq 500
D	Ribo-Zero Plus	Proprietary enzymatic depletion	Illumina Stranded Total RNA Prep Kit	Random hexamers	Illumina NextSeq 550/200
E^*b*^	microbExpress	Oligo capture and magnetic bead separation	NEBNext sRNA Library Prep Set for Illumina	Adapters	Illumina NovaSeq 6000

^*a*^ For sequencing pipeline details of airway epithelial cell models, see Dataset S1.

^*b*^ Samples were sequenced at different sequencing facilities (see Materials and Methods for more details).

**Fig 2:**
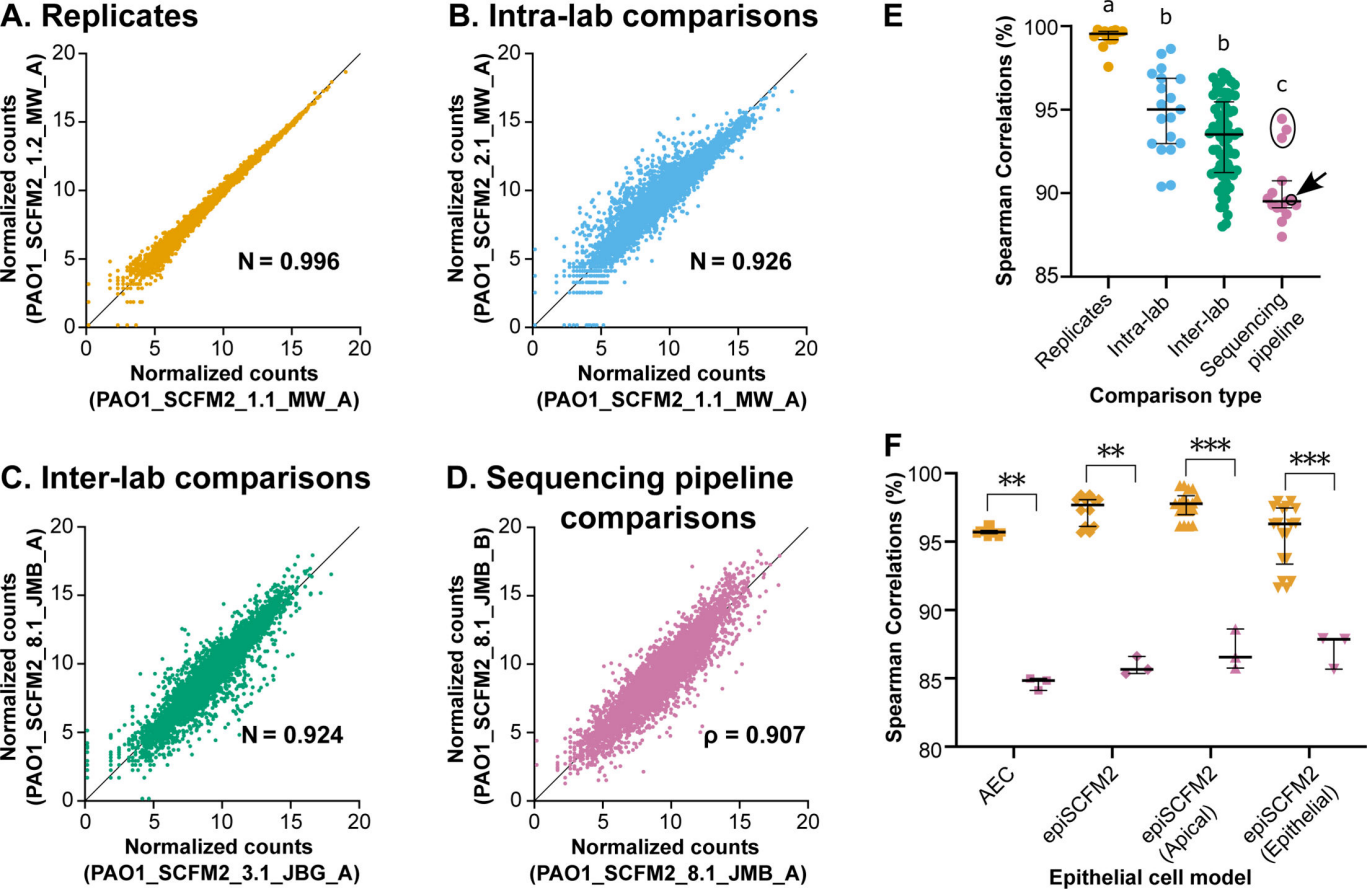
RNA-seq data are highly reproducible between labs and sequencing pipelines. (**A–D**) Representative plots of VST-normalized gene expression between two SCFM2 datasets for each of the four comparison categories. Spearman correlation coefficients (*ρ*) are given in each plot. Representative correlation plots show the median correlation coefficients of each comparison category. (**E**) Spearman correlation coefficients plotted by comparison type for SCFM2. Each data point represents the correlation coefficient for a pair of data sets. Different letters above comparison types indicate statistically significantly different medians between those types based on a Kruskal-Wallis test followed by a Dunn’s multiple comparisons test (a and b: *P* < 0.05 for replicates vs intra-lab comparisons and *P* < 0.0001 for replicates vs inter-lab comparisons; a–c: *P* < 0.0001, b and c: *P* < 0.001 for intra-lab comparisons vs pipeline comparisons and *P* < 0.01 for inter-lab comparisons vs pipeline comparisons). No significant difference was found between intra-lab and inter-lab comparisons. The arrow points to the correlation coefficient between data sets that were sequenced with sequencing pipelines B and E (outlined in black for clarity), which were sequenced at the same site with the same Illumina platform but had different ribosomal RNA depletion and library preparation procedures (see Materials and Methods and Dataset S1). The three circled data points correspond to correlation coefficients for paired data sets sequenced with sequencing pipelines B and D. (**F**) Spearman correlation coefficients plotted by comparison type for airway epithelial cell models. Data point shape represents model, and data point color represents replicate sample comparisons (orange) or sequencing pipeline comparisons (pink). AEC, CF airway epithelial cell model. epiSCFM2, CF airway epithelial cell-SCFM2 model. Asterisks represent statistically significantly different rank sums in correlation coefficients between the two comparison types based on a Mann-Whitney *U* test (**P < 0.01; ***P < 0.001). In all panels, data points are color-coded by comparison type (orange: replicates, blue: intra-lab comparisons, green: inter-lab comparisons, and pink: sequencing pipeline comparisons). In panels E and F, medians ± interquartile range are shown.

To examine variance in transcriptomes, we focused on gene expression in *P. aeruginosa* strain PAO1 grown *in vitro* in SCFM2. To control for the known variance in PAO1 strains (21), the same stock culture was used (22); however, the SCFM1 and SCFM2 media were prepared independently in each of our three laboratories. *P. aeruginosa* gene expression in SCFM2 was normalized by variance stabilizing transformation (VST), and Spearman correlation coefficients were (*ρ*) calculated. Samples were classified in four ways: (i) replicates—samples prepared in parallel in the same lab and sequenced with the same sequencing pipeline (Fig. 2A), (ii) intra-lab comparisons—samples prepared in the same lab on different days and sequenced with the same sequencing pipeline (Fig. 2B), (iii) inter-lab comparisons—samples prepared in different laboratories and sequenced with the same sequencing pipeline (Fig. 2C), and (iv) sequencing pipeline comparisons—the same sample that was sequenced with different sequencing pipelines (Fig. 2D).

First, we aimed to understand how sample preparation within and between labs affects transcriptional profiles. Transcriptional profiles correlated differently depending on the type of comparison (Fig. 2A through E). Replicate data sets were highly correlated, with *ρ* ranging from 97.6% to 99.8% (median = 99.4%) (Fig. 2A and E). Intra-lab comparisons had a larger range at 90.4%–98.6% (median = 95.0%) (Fig. 2B and E). Finally, inter-lab comparisons had a slightly larger range and slightly lower median *ρ* than intra-lab comparisons at 88.0%–97.2% (median = 92.7%) (Fig. 2C and E), but inter-lab and intra-lab comparisons were not significantly different (Kruskal-Wallis statistic = 54.80, *P* = 0.3580). The median correlation coefficient among replicates was significantly higher than the median correlation coefficients of inter-lab and intra-lab comparisons (Kruskal-Wallis statistic = 54.80, *P* < 0.0001 followed by a Dunn’s multiple comparisons test: *P* < 0.05 for replicates vs intra-lab comparisons, *P* < 0.001 for replicates vs inter-lab comparisons). These data indicate that RNA-seq data are reproducible across laboratories and can be validly compared (17).

To understand how sequencing pipeline contributes to variance in RNA-seq data, we compared data sets generated using the same sample but sequenced with different sequencing pipelines (Tables 1 and 2; Table S1) (Fig. 2D through F). In SCFM2 datasets, sequencing pipeline comparisons showed significantly more expression variance than intra-lab comparisons (median *ρ* = 89.1%; Kruskal-Wallis statistic = 54.80, *P* < 0.0001 followed by a Dunn’s multiple comparisons test: *P* < 0.001). However, Spearman correlation coefficients between sequencing pipelines (range: 87.4%–94.5%) did overlap with the range of both intra-lab and inter-lab correlation coefficients (Fig. 2E). Furthermore, for SCFM2 samples, some sequencing pipelines had similar expression variance compared to the inter-lab and intra-lab variance—expression from pipelines B and D (circled points in Fig. 2E) correlated similarly to inter-lab and intra-lab median *ρ* values (*ρ*: 93.3%–94.5%). In contrast, other pipeline pairs correlated slightly less well (*ρ*: 87.4%–90.7%). In addition, in four different airway epithelial cell models of *P. aeruginosa* infection in CF, the rank sum of *ρ* of sequencing pipeline comparisons was 84.8%–87.9%, similar to SCFM2 but lower than the rank sum of *ρ* of replicate samples sequenced with the same pipeline (95.3%–97.8%) (Mann-Whitney *U* test, *P* < 0.05 for all) (Fig. 2F). Thus, while some sequencing pipelines result in more similar results than others, Spearman correlations ≥ 84.8% indicate that the sequencing pipelines we examined all resulted in similar expression profiles.

### Expression variance between sequencing pipelines is driven by library preparation

The expression differences we observed between sequencing pipelines (Fig. 2) raise the question of how sequencing pipelines introduce variance. The sequencing pipelines we used differed in library preparation approaches (including ribo-depletion, the order of cDNA library construction steps, priming sites for reverse transcription of mRNA, and size selection method), sequencing facility, and Illumina sequencing platforms (NovaSeq 6000, NextSeq 500/550, or NextSeq 2000) (Table 2; Dataset S1). Sequencing platform is not likely to be a large source of variance because all platforms we employed use one of two variations on Illumina’s proprietary 2-channel sequence by synthesis chemistry. Furthermore, one of the sequencing pipelines we used (pipeline A) included samples that were sequenced by two different Illumina platforms (Table 2; Table S1), and *P. aeruginosa* expression profiles correlated well between the two platforms (median *ρ* = 93.5%) (Fig. S1). We thus hypothesized that library preparation was responsible for expression variance in sequencing pipelines. Two SCFM2-grown PAO1 data sets supported our hypothesis. These data sets were generated from the same RNA sample, underwent different library preparation procedures, but were sequenced in parallel at the same sequencing facility (sequencing pipelines B and E) (Tables 1 and 2; Dataset S1). The Spearman correlation coefficient between these samples was 89.6% (arrow in Fig. 2B, data point highlighted with a black outline). Furthermore, a principal component analysis found the data set that was sequenced with pipeline E clustered with samples sequenced with pipeline A, while the same sample sequenced with pipeline B clustered with other pipeline B samples (Fig. S2). The fact that pipelines A and E use the same library preparation suggests that different library preparation approaches were responsible for the divergence in transcriptional profiles between pipelines B and E. Thus, it is important for investigators to consider that library preparation may contribute to differences across samples (18, 23–25).

### Explaining expression differences between pipelines A and B

Which step in the library preparation is responsible for the variance observed between the sequencing pipelines? To answer this question, we focused on pipelines A and B, for which we have the most SCFM2 datasets. Library preparation in these pipelines differs in two ways (Fig. S3; Table 2): (i) pipeline A depletes ribosomal RNAs (rRNAs) through hybridization to oligonucleotides and separation with magnetic beads, while pipeline B depletes rRNAs by enzymatic digestion of abundant DNA/RNA hybrid molecules following reverse transcription; and (ii) pipeline A uses a small RNA (sRNA) library preparation kit, while pipeline B uses a standard mRNA library preparation kit that targets transcripts between 150 and 900 bp. As enzymatic rRNA depletion is prone to off-target activity (26) and the sRNA library kit targets smaller RNAs, we hypothesized that the differences in library preparation may lead to differences in the detection of highly expressed genes and/or small transcripts.

**Fig 3:**
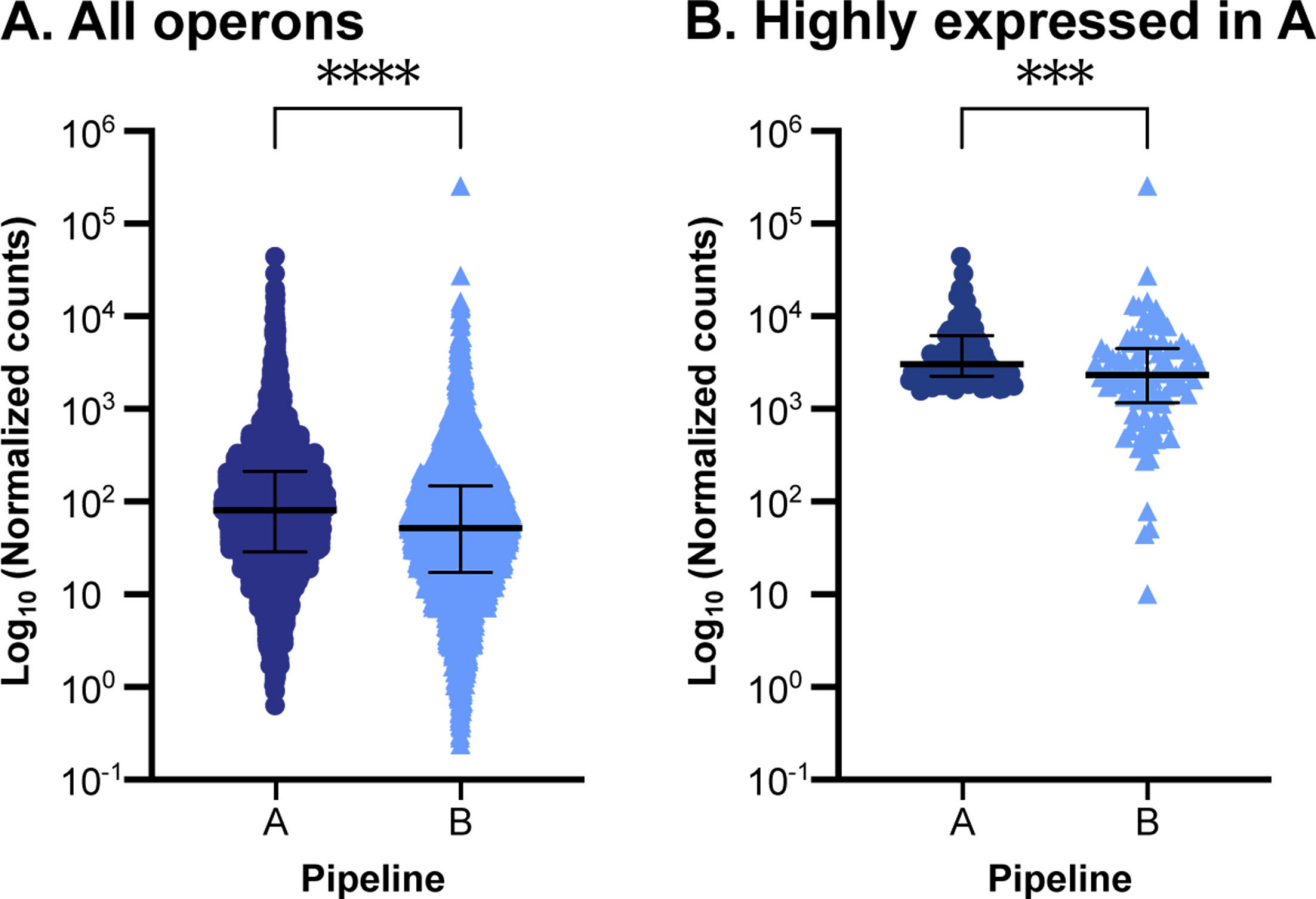
Operons are more highly expressed in pipeline A than in pipeline B. (**A**) Average Log_10_ (TPM-normalized expression) of all *P. aeruginosa* operons in sequencing pipelines A or B. Shape and color denote the pipeline. (**B**) Average Log_10_ (TPM-normalized expression) in pipelines A and B of *P. aeruginosa* operons highly expressed in pipeline A. Highly expressed operons were determined by ranking each operon’s average TPM-normalized expression in pipeline A and calculating the inflection point of the curve (Fig. S4A). Median ± interquartile range is shown. Significance was determined using a Mann-Whitney *U* test. ****P < 0.0001; ***P < 0.001.

**Fig 4:**
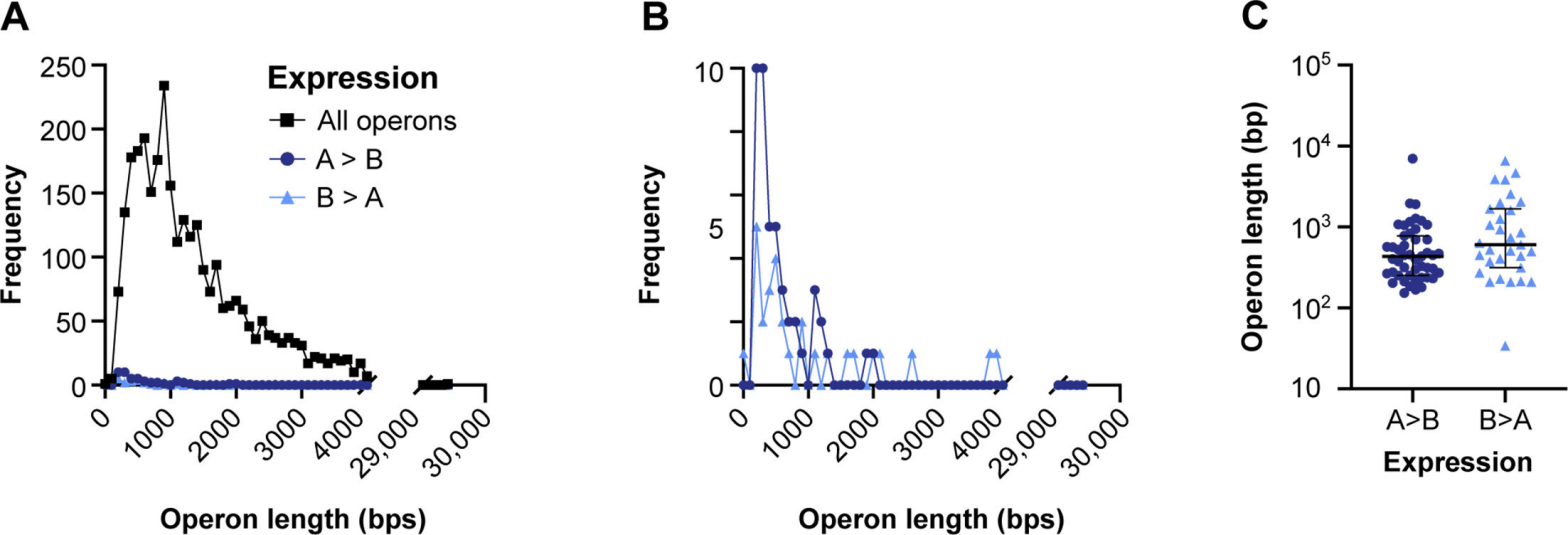
Relationship between operon length and expression variance due to size selection. (**A**) Frequency of operons as a function of length for all operons and operons with the highest expression difference between pipelines A and B (see Fig. S6). (**B**) Frequency of operons with highest expression difference between pipelines A and B with *y* axis adjusted. Color and shape of data points denote the sequencing pipeline where the operons were more highly expressed. Bin widths for operon length in frequency distributions were set to 100. (**C**) Average lengths of operons with the high expression differences between pipelines A and B. Median and interquartile range of operon length for set of operons are shown. Significance between pipelines was determined by a Mann-Whitney *U* test (*P* = 0.07).

- *Pipeline B depletes highly expressed operons.* If pipeline B depletes highly abundant mRNAs, then we expect to see that normalized transcript counts would be lower in pipeline B than in pipeline A. We tested this using *P. aeruginosa* operons because the library preparation protocol of pipeline B does not fragment RNA transcripts before enzymatically depleting rRNA. First, we normalized *P. aeruginosa* operon expression for operon length and coverage using transcripts per million (TPM) and compared the number of normalized counts assigned to each operon in both pipelines (Fig. 3A). Operons had significantly fewer normalized counts in pipeline B (median = 51.98) than pipeline A (median = 81.35; Wilcoxon matched-pairs rank test: *P* < 0.0001), supporting our speculation. To further understand if these differences were driven by highly abundant operons, we selected the 93 operons with the highest normalized counts in pipeline A (see Fig. S4A) and compared the number of normalized counts assigned to these operons between the two pipelines. The number of normalized counts assigned to these 93 operons was significantly lower in pipeline B (median = 2,308.74) compared to pipeline A (median = 3,055.19; Wilcoxon matched-pairs rank test: *P* < 0.001) (Fig. 3B). We also found similar results by analyzing these data at the gene level (Fig. S5). Taken together, these data demonstrate that sequencing pipeline B depletes transcripts (operons) that are highly detected by pipeline A, supporting how library preparation contributes to the expression variance between the two pipelines (Fig. 2).
- *Pipeline A sequences small transcripts*. If pipeline A selects for smaller transcripts due to the use of the small RNA kit, then we would expect that pipeline A had increased detection of short transcripts (operons) compared to pipeline B. This increased abundance of short operons in pipeline A would result in a more right-skewed distribution of operon frequency vs length in pipeline A than in pipeline B. To test this, we first examined the frequency of all *P. aeruginosa* operons as a function of length (Fig. 4A). The distribution of all operons as a function of length was right-skewed (skewness = 4.081), with the most frequent operon length at 900 bp and an extended tail of longer operons. Then, we compared this distribution to that of operons with large differences in normalized counts between pipelines A and B (see Fig. S6A and B). The frequency vs length distribution for operons that were more highly expressed in pipeline A was more right skewed than those more highly expressed in pipeline B (8.212 and 6.535, respectively), suggesting that pipeline A resulted in increased detection of smaller operons relative to pipeline B (Fig. 4B). We also found that the lengths of the operons with higher expression in pipeline A trended smaller than those with higher expression in pipeline B, but this was not significantly different using a Mann-Whitney *U* test (*P* = 0.0718) (Fig. 4C). When we performed the same analyses on *P. aeruginosa* genes, we found a similar pattern (Fig. S6C, D and S7), and the genes with higher expression in pipeline A were significantly smaller than those with higher expression in pipeline B (Fig. S7). These data indicate that the sRNA kit used in pipeline A may select for smaller transcripts than pipeline B, further demonstrating how library preparation contributes to expression variance between sequencing pipelines.
- *Differential sequencing of small, noncoding RNAs*. We additionally asked if small, noncoding RNAs were better captured in sequencing pipeline A than B, as these genes were not included in our analyses above. We examined the average TPM-normalized expression of 93 sRNAs ranging from 28- to 150-bp long in pipelines A and B, including sRNAs that were recently identified by Trouillon et al. (27). We compared the expression of sRNAs that were 28–150 bp, 28–100 bp, or 28–50 bp (Fig. S8A through C). We found no significant difference in expression between pipelines in sRNAs of any of the three size categories using a Wilcoxon matched-pairs rank test. Thus, pipeline A does not enhance the detection of sRNAs compared to pipeline B.

### Sequencing pipelines A and B agree in the detection of differentially expressed genes

**Fig 5:**
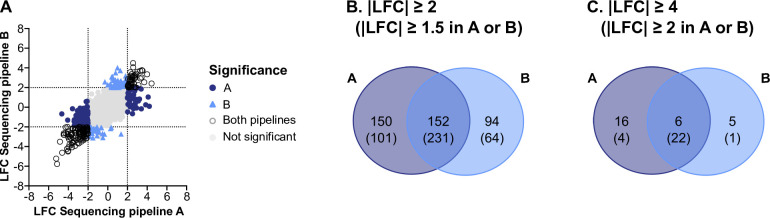
Sequencing pipelines detect similar differentially expressed genes. (**A**) Average Log_2_(fold change) for each gene between SCFM2 and epiSCFM2 using samples that were prepared in the same lab and sequenced with both pipelines A and B (Dataset S1). Significance, defined as adjusted *P* value < 0.001 and |LFC| ≥ 2, is indicated by color and shape (see key). The significance category “Not significant” includes genes with adjusted *P* values > 0.001 and genes with |LFC| < 2. (**B**) Venn diagram showing overlap in differentially expressed genes (DEGs between sequencing pipelines at a cutoff of |LFC| ≥ 2. Shown in parentheses are the number of DEGs detected by both pipelines at |LFC| ≥ 2 combined with DEGs that are detected by one pipeline at |LFC| ≥ 2 and one pipeline at |LFC| ≥ 1.5 to include DEGs just below the cutoff. (**C**) Venn diagram showing overlap in DEGs between sequencing pipelines at a cutoff of |LFC| ≥ 4. Shown in parentheses are the number of DEGs detected by both pipelines at |LFC| ≥ 4 combined with DEGs that are detected by one pipeline at |LFC| ≥ 4 and one pipeline at |LFC| ≥ 2 to include DEGs just below the cutoff.

Perhaps the most common use of RNA-seq data is to determine if and how genes are differentially expressed between different treatments or conditions. The variance in gene expression we observed between sequencing pipelines A and B (Fig. 2E and F) prompted us to ask whether it would influence the detection of differentially expressed genes (DEGs). For each pipeline, we measured differential expression in *P. aeruginosa* genes between SCFM2 and the epiSCFM2 model, controlling for sample number in each condition. To directly compare pipelines, all SCFM2 and epiSCFM2 samples used were prepared in the same laboratory and sequenced with both pipelines A and B (see Dataset S2 for samples included in each analysis). After calculating differential expression between models, we compared the Log_2_ (fold change) (LFC) of all genes between sequencing pipelines (Fig. 5A). Overall, the LFC values representing differential expression between SCFM2 and epiSCFM2 correlated well between the two pipelines. Using a |LFC| cutoff of 2 for differential expression, each pipeline detected similar numbers of DEGs (A: 302, B: 246, adjusted *P* ≤ 0.001). Of these DEGs, 152 were detected by both pipelines (Fig. 5B), resulting in 150 genes uniquely detected by pipeline A and 94 genes uniquely detected by pipeline B. Importantly, many genes that were detected by only one pipeline had borderline LFC values in the other pipeline (Fig. 5A). When we relaxed the |LFC| cutoff to 1.5, we found a total of 244 additional DEGs detected by at least one pipeline, of which 79 DEGs previously only detected by one pipeline were now detected by the other pipeline, increasing the number detected by both pipelines from 152 to 231, leaving 101 DEGs uniquely detected by pipeline A and 64 uniquely detected by pipeline B. Thus, 231 DEGs were detected by both pipelines, albeit 79 with borderline LFCs slightly below the |LFC| cutoff of 2 in one of the pipelines. We also considered a more stringent |LFC| cutoff of 4 to identify genes with the highest expression differences between SCFM2 and epiSCFM2. We found that pipeline A detected 22 DEGs and pipeline B detected 11 DEGs, with 6 DEGs detected by both pipelines, 16 DEGs uniquely detected by pipeline A, and 5 DEGs uniquely detected by pipeline B (Fig. 5C). When we relaxed the |LFC| cutoff to 2, we found a total of 27 additional DEGs, of which 16 DEGs previously only detected by one pipeline were now detected by the other, increasing the number of DEGs detected by both pipelines from 6 to 22, leaving 4 DEGs uniquely detected by pipeline B and 1 uniquely detected by pipeline B. Thus, 22 highly expressed DEGs were detected by both pipelines, albeit 16 DEGs with borderline LFCs slightly below the |LFC| cutoff of 4 in the other pipeline. These results indicate that despite some discrepancies in DEG detection between sequencing pipelines A and B, overall, these two sequencing pipelines reproduce highly similar results in a differential expression analysis between different conditions.

### Conclusions, implications, and recommendations

Reproducibility of sequencing pipelines (Fig. 1, 2, and 5) is especially relevant because as kits are discontinued, technologies develop, and new platforms arise, sequencing facilities may change their pipeline (e.g., pipelines C and D of this study—see Materials and Methods). Thus, even labs that use the same sequencing facility may be required to compare across sequencing pipelines. Because RNA-seq reproducibility studies typically focus on eukaryotes, we set out to examine sources of variance in bacterial RNA-seq data. Our finding that bacterial RNA-seq data generated by different labs and sequenced with different sequencing pipelines are reproducible (Fig. 2) demonstrates that gene expression data from different studies can indeed be robustly compared. Sequencing pipelines introduced the most variance (Fig. 2E and F), due, in part, to differences in ribo-depletion and size selection (Fig. 3 and 4; Fig. S2 to S8).

While our data are largely encouraging, it also reveals that some sequencing pipelines diverge more than others in the expression of specific genes. For example, sequencing pipelines A and B had an overall correlation of 89.5% in SCFM2 samples but specifically detect an order of magnitude difference in the average normalized expression of the *P. aeruginosa* sRNA *sicX* (PA1414) (14,865.76 in pipeline A vs 3,216.50 in pipeline B; Dataset S2). *sicX* is a regulator of the chronic-to-acute transition in infections (28) and thus highly relevant to chronic infections. *sicX* is also a short and highly abundant transcript, the two factors we identified that differentiate pipelines A and B, demonstrating that for certain genes, validation such as by qRT-PCR and/or northern blot is necessary. In addition, RNA-seq data interpretation and study comparison would be strengthened by the development and implementation of standard reporting guidelines of experimental methods, much like other fields have implemented to improve consistency and reproducibility (29–32). We propose that RNA-seq studies should report details on all aspects of workflow, including sample preparation, collection, preservation and storage, RNA extraction and treatment, library preparation, sequencing platform, computational analysis, programs, and code.

## MATERIALS AND METHODS

### Strains, media, and *in vitro* growth conditions

*P. aeruginosa* MPAO1 (22) was used for *in vitro* experiments with SCFM2. SCFM1 and SCFM2 were prepared as described (11, 19). Culture conditions are given in Dataset S1. Briefly, *P. aeruginosa* was grown on Brain Heart Infusion agar and incubated overnight at 37°C. For all but two SCFM2 samples (described below), a single colony was inoculated into SCFM1 for overnight growth at 37°C, shaking at 200–250 rpm. The following day, overnight cultures were inoculated into SCFM2 to an OD_600_ of 0.05 in two separate 4-well chambered coverglass plate (Lab-Tek) and grown statically for 6 hours at 37°C, as previously described (17). For two SCFM2 samples, the overnight culture from a single colony was inoculated into SCFM2 to an OD_600_ of 0.05 in two separate 4-well chambered coverglass plate and then each was grown statically for 6 hours at 37°C (technical replicates). All cultures were preserved in RNAlater (ThermoFisher) and stored at −80°C prior to RNA extraction.

### CF airway epithelial cell models

The AEC and epiSCFM2 models were performed as previously described (17, 18) using air-liquid interface differentiated immortalized homozygous ΔF508/ΔF508-CFTR CFBE41o- human bronchial epithelial cells (obtained from J.P. Clancy, Cincinnati Children’s Hospital).

### RNA extraction

RNA was extracted from *in vitro* samples and airway epithelial cell models as described (17, 18) with modifications. Briefly, after thawing, samples were pelleted, RNAlater was removed, and pellets were resuspended in RNase-free TE buffer containing lysozyme, or lysozyme and lysostaphin. Samples were transferred to pre-filled bead beating tubes (MIDSCI) and mechanically lysed by bead beating for 30 seconds and then incubated at 37°C for 30 minutes for enzymatic lysis. RNA-Bee or RNA Stat-60 (Amsbio) was added, and samples were bead beaten 3× for 30 seconds, placing on ice in between each round. Chloroform was added to each sample, and samples were vigorously shaken and centrifuged to separate phases. The aqueous phase was removed to a new tube, and the RNA was precipitated with isopropanol. RNA samples were treated with DNase I (Promega) in solution, cleaned up with RNA Bee or Stat-60 using the above steps (following the lysis step), and quantified using a nanodrop. RNA quality was assessed with a Bioanalyzer 2100 (Agilent).

### RNA sequencing pipelines

Samples treated with DNase I were sequenced with at least one of five RNA sequencing pipelines (Table 1), A, B, C, D, and E. “Sequencing pipeline” refers collectively to rRNA depletion, cDNA library preparation, size selection, and Illumina sequencing. Sequencing pipeline details for each sample are provided in Dataset S1.

#### Sequencing pipeline A

For all samples (*in vitro* and airway epithelial cell models), sequencing libraries were constructed as previously described (17) with modifications. For *in vitro* (SCFM2) samples, ribosomal RNA was first depleted using the Invitrogen MICROBExpress Bacterial mRNA Enrichment Kit and then mRNA was fragmented with the NEBNext Magnesium fragmentation module (New England Biosciences), and sequencing libraries were prepared using the NEBNext Small RNA Library Prep Kit (New England Biosciences). For airway epithelial cell samples, total RNA was fragmented using the NEBNext Magnesium fragmentation module (New England Biosciences) if the RNA integrity number (RIN) was greater than 4.0, then libraries were constructed using the NEBNext Small RNA Library Prep Kit (New England Biosciences). Samples with RIN ≤ 4.0 proceeded directly to library construction without fragmentation. rRNA was depleted using the Qiagen QIAseq FastSelect Kits (HMR and 5S/16S/23S) following the 5′ SR adapter ligation step, and library construction was continued after rRNA depletion. For both sample types, library construction was followed by size selection on a 5% TBE polyacrylamide gel to remove adapter dimers. Samples were sequenced at the Molecular Evolution Core at the Georgia Institute of Technology on an Illumina NextSeq 500 using 75-bp single-end runs or on an Illumina NovaSeq 6000 using 100-bp single-end runs.

#### Sequencing pipeline B

Total RNA treated with DNase was sent to Zymo Research (Irvine, CA, USA) for sequencing with their commercial pipeline. Briefly, 500 ng of total RNA was used to construct sequencing libraries. rRNA was removed as described by Bogdanova et al. (33), and libraries were prepared using the Zymo-Seq RiboFree Total RNA Library Prep Kit (Zymo) following the manufacturer’s protocol. Samples were sequenced on an Illumina NovaSeq 6000 using paired-end, 150-bp runs.

#### Sequencing pipelines C and D

Total RNA treated with DNase was sent to the Microbial Genome Sequencing Center (Pittsburgh, PA, USA) for sequencing with one of two commercial pipelines. For sequencing pipeline C (used for two SCFM2 samples), rRNA was depleted using the QIAseq FastSelect 5S/16S/23S kit (Qiagen), and libraries were prepared using the QIAseq Total Stranded RNA library prep kit (Qiagen). Samples were sequenced on an Illumina NextSeq 500 using a single-end, 75-bp run. For the samples sequenced with pipeline D, rRNA was depleted using Ribo-Zero Plus (Illumina), and libraries were constructed using the Illumina Stranded Total RNA prep kit. Samples were sequenced on an Illumina NextSeq 550 using single-end, 75-bp runs or on an Illumina NextSeq 2000 using single-end, 50-bp runs.

#### Sequencing pipeline E

One SCFM2 sample was subjected to a hybrid pipeline. For this sample, an RNA-seq library was prepared as described above for sequencing pipeline A (rRNA depletion with MICROBExpress kit, library prepared with NEBNext sRNA library prep kit and size selection to remove adapter dimers). The library was then sent to Zymo Research for sequencing on an Illumina NovaSeq 6000 (pipeline E) alongside a paired library prepared by Zymo using their standard library prep kit (pipeline B) from the same source RNA.

### RNA-seq data analysis

RNA-seq data were analyzed as described (17). Briefly, Illumina adapters were trimmed from RNA-seq reads in cutadapt (version 3.4) (34) using a minimal read length threshold of 22 bp. After checking read quality in FastQC (version 0.11.9), reads were mapped to the genomes of 105 non-*P*. *aeruginosa* decoy strains (as described in reference [17]) using bowtie2 (version 2.4.2) (35). Reads that failed to map to decoy genomes were subsequently mapped to the PAO1 reference genome (NC_002516.2, NCBI Assembly: GCF_000006765.1) using bowtie2. Reads were assigned to PAO1 coding sequences, operons, or small noncoding RNAs and counted in FeatureCounts (version 2.0.1) (36), with reads assigned to overlapping meta-features (if applicable) and strandedness specified (strandedness information is provided in Dataset S1). Operon counts were generated with a custom annotation file for *P. aeruginosa* operons. sRNA counts were generated using a custom annotation file that contained 44 sRNAs present in the original annotation file (GCF_000006765.1) as well as 128 newly described sRNAs (27). Read counts for each sample were combined into count matrices for use in downstream analyses. We reduced the gene counts data set to include only the 5,147 genes in the core *P. aeruginosa* gene set, as determined by Lewin et al. (17).

#### Spearman correlations

Spearman correlation coefficients were calculated for pairs of SCFM2 data sets and pairs of data sets from each epithelial cell model. For each sample type, counts for all core coding sequences were normalized by VST in the R package DESeq2 (37), and Spearman correlation coefficients were calculated in the R package psych (38). Correlation coefficients were plotted in Prism 9 (GraphPad). For SCFM2, a Kruskal-Wallis test (followed by a corrected Dunn’s multiple comparisons test) was performed to determine if median correlation coefficients for each comparison type were statistically significantly different from one another. For epithelial cell models, a Mann-Whitney *U* test was used for each model to determine if the rank sum of correlation coefficients between replicates and sequencing pipeline comparisons were statistically significantly different.

#### Testing hypotheses for variance observed between pipelines A and B

To test two *a priori* hypotheses for how library preparation approaches between sequencing pipelines A and B introduced variance in the overall expression of SCFM2 samples sequenced with both pipelines, resulting in paired RNA-seq data sets. We normalized raw counts for each data set by TPM by dividing counts for each gene, operon, or sRNA by its length in kilobases to give reads per kilobase (RPK) and then dividing RPK values by a scaling factor (sum of all RPK values by 1,000,000).

#### Testing for depletion of abundant transcripts in pipeline B

We determined which genes and operons were the most abundantly expressed in pipelines A and B by plotting average TPM-normalized counts against operons or genes ranked by expression, from highest to lowest. We then used the R package inflection to calculate the inflection point of each curve. Operons and genes with higher expression than the expression corresponding to the inflection point were considered “abundant.” Next, we compared TPM-normalized counts between pipelines A and B for all operons or core genes, and for operons or core genes that were abundant. Significance was tested in Prism 9 using a Mann-Whitney *U* test.

#### Testing for selection for small transcripts in pipeline A

We determined which operons or genes had the highest difference in expression between pipelines A and B by plotting the difference in average, TPM-normalized expression against length in base pairs. We chose operons and genes with a difference of at least 1,000 TPM-normalized counts because this number captured mostly small transcripts and excluded the very long operons and genes with a very small expression difference, while still maintaining an appropriate sample size for comparison. We then generated frequency distributions against transcript length for all transcripts and transcripts that were highly expressed in one of the two pipelines. In addition to examining the distribution of transcripts in these different categories by length, we also compared gene length between pipelines for the three categories using column plots. Statistical significance was determined for the column plots in Prism 9 with a Mann-Whitney *U* test.

#### Testing for selection of sRNAs in pipeline A

sRNAs were not included in our above analysis of coding sequence transcriptional profile variance, so we performed a separate analysis to test the hypothesis that pipeline A selects for sRNAs. We compared average TPM-normalized expression between pipelines A and B for four categories of sRNAs: (i) all sRNAs, (ii),sRNAs ≤ 150 bp, (iii) sRNAs ≤ 100 bp, and (iv) sRNAs ≤ 50 bp. Significance in expression between pipelines of sRNAs in these four categories was determined in Prism 9 using a Wilcoxon matched-pairs rank test.

#### Differential expression analyses

To compare differential expression results between sequencing pipelines A and B, differential expression was measured between SCFM2 and the epiSCFM2 model in DESeq2 for each sequencing pipeline. To isolate sequencing pipeline as a variable, we used the three epiSCFM2 and SCFM2 samples prepared in the lab of Jennifer Bomberger, which had been sequenced with both pipelines (Dataset S1). Differential expression was measured in DESeq2 with log_2_ (fold change) shrinkage (apeglm) (39). LFC correlation was plotted for each pair of sequencing pipelines in Prism9 (GraphPad).

## Data Availability

Raw RNA-seq reads not used in previously published studies were deposited in the NCBI Sequence Read Archive (SRA) database under BioProject PRJNA1082245. SRA accessions for raw reads from new data sets are listed in Dataset S1. SRA accessions and references for raw reads from previously published data sets included in this study are listed in Dataset S1.

## SUPPLEMENTAL MATERIAL

Dataset S1 spectrum.01513-24-s0001.xlsx Sample information.

Dataset S2 spectrum.01513-24-s0002.xlsx Datasets used in differential expression analyses between SCFM2 and epiSCFM2 and differential expression results for samples sequenced with pipelines A and B.

Supplemental figures spectrum.01513-24-s0003.pdf Fig. S1 to S8.

ASM does not own the copyrights to Supplemental Material that may be linked to, or accessed through, an article. The authors have granted ASM a non-exclusive, world-wide license to publish the Supplemental Material files. Please contact the corresponding author directly for reuse.
