# Supplementary material for: RNA-seq reproducibility of Pseudomonas aeruginosa in laboratory models of cystic fibrosis
Source: Microbiol Spectr. 2024 Dec 3;13(1):e01513-24. doi: 10.1128/spectrum.01513-24 (PMC11705926; doi:10.1128/spectrum.01513-24)
Supplement: Supplemental figures — Fig. S1 to S8. [file spectrum.01513-24-s0003.pdf]

Supporting Information

RNA-seq reproducibility of *Pseudomonas aeruginosa* in laboratory models of cystic fibrosis

Duncan, Rebecca P.[1,2,a,†], Lewin, Gina R.[2,3,b,†], Cornforth, Daniel M.[2,3], Diggle, Frances L.[2,3], Kapur, Ananya[4], Moustafa, Dina A.[1,2], Hilliam, Yasmin[4], Bomberger, Jennifer M.[4,c], Whiteley, Marvin[2,3,*], and Goldberg, Joanna B.[1,2,*]

[†]These authors contributed equally

Author affiliations:

[1]Division of Pulmonary, Asthma, Cystic Fibrosis, and Sleep, Department of Pediatrics, Emory University School of Medicine, Atlanta, GA, USA

[2]Emory-Children's Cystic Fibrosis Center, Atlanta, GA, USA

[3]School of Biological Sciences and Center for Microbial Dynamics and Infection, Georgia Institute of Technology, Atlanta, GA, USA

[4]Department of Microbiology and Molecular Genetics, University of Pittsburgh, Pittsburgh, PA, USA

[a]Current address: Division of Viral Diseases, Tanaq Management Services, contracting agency to the Polio and Picornavirus Branch, Centers for Disease Control and Prevention, Anchorage 99503, AK, USA

[b]Current address: Department of Pathology, Center for Global Health and Diseases, School of Medicine, Case Western Reserve University, Cleveland, OH, USA

[c]Current address: Department of Microbiology and Immunology, Geisel School of Medicine, Dartmouth College, Hanover, NH, USA

*Corresponding authors:

Joanna B. Goldberg
Department of Pediatrics
Emory University School of Medicine
Emory-Children's Cystic Fibrosis Center
E-mail: joanna.goldberg@emory.edu

Marvin Whiteley
School of Biological Sciences, Georgia Institute of Technology
Emory-Children's Cystic Fibrosis Center
E-mail: mwhiteley3@gatech.edu

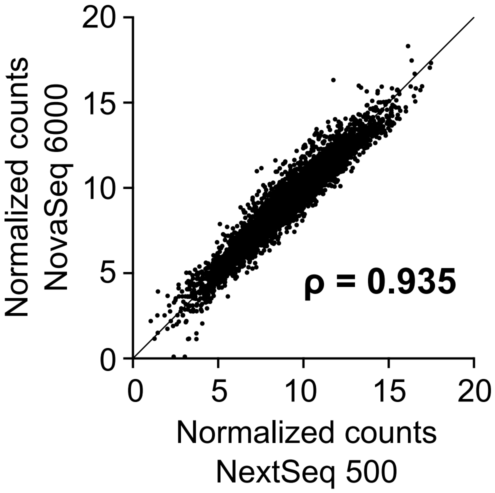

Figure S1. ***P. aeruginosa*** **gene expression profiles correlate well between Illumina sequencing platforms.** Average variance stabilizing transformation (VST) normalized counts for samples sequenced on the Illumina NovaSeq 6000 platform are plotted against average VST normalized counts for different samples sequenced on the Illumina NextSeq 500 platform (see Dataset S1). All samples were sequenced with sequencing pipeline A. The median Spearman correlation coefficient for all pairwise comparisons of datasets used in this plot is shown (ρ).

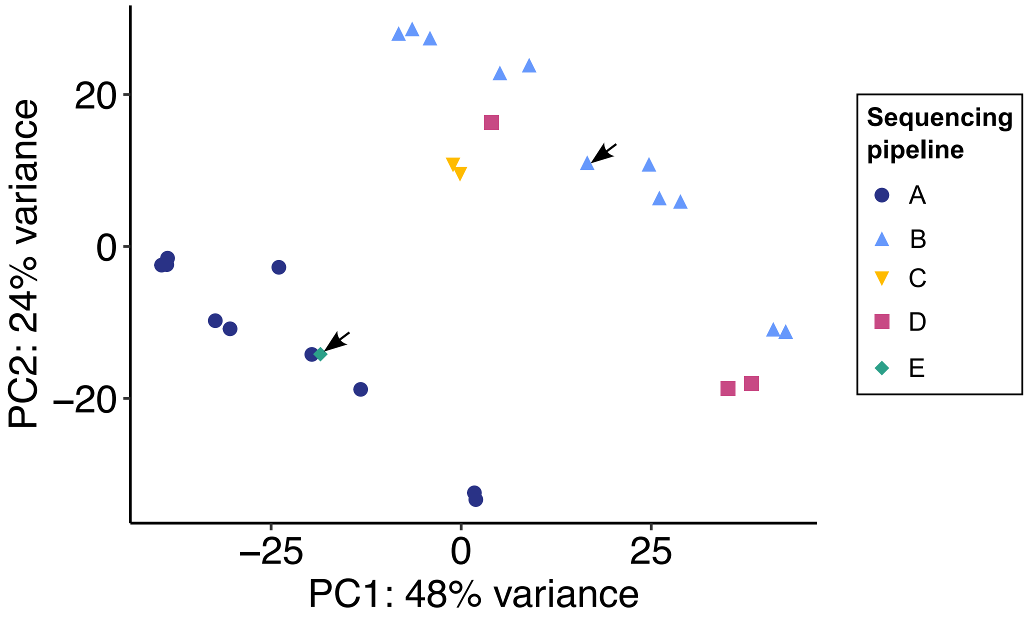

Figure S2. SCFM2 datasets cluster by sequencing pipeline in a principle component analysis. A principle component analysis of all SCFM2 RNA-seq datasets was conducted in the R package DESeq2 based on the 500 genes with the most variance out of the 5147 core *P. aeruginosa* gene set used in this study. Each data point represents a different RNA-seq dataset, and sequencing pipeline is indicated by data point colors and shapes according to the key. Arrows indicate the sample with libraries prepared using two different kits but sequenced at the same sequencing facility (Dataset S1).

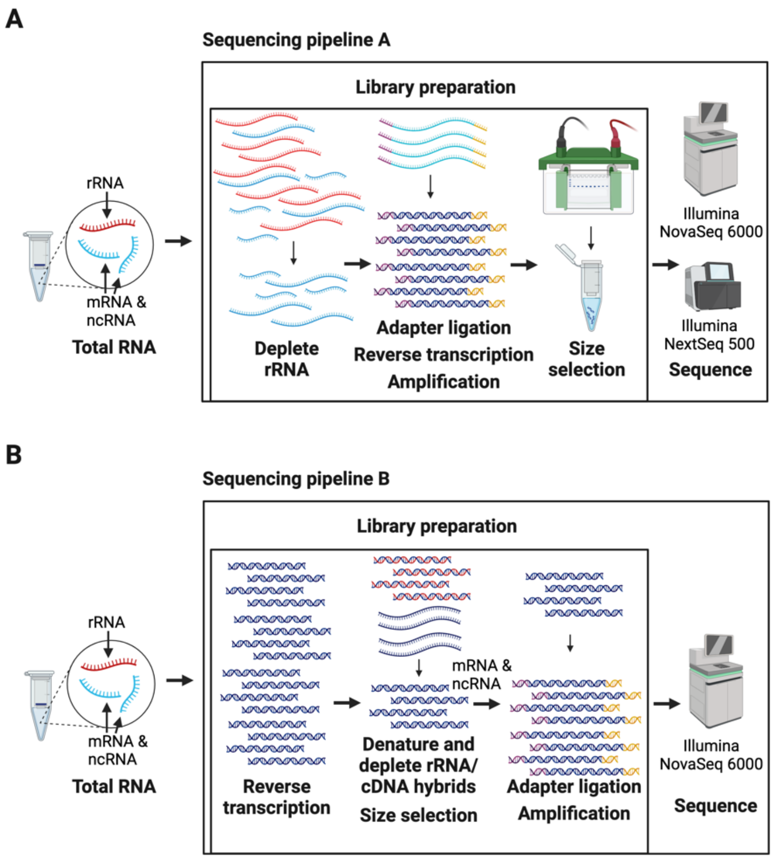

Figure S3. Sequencing pipelines A and B use different library preparation methods. Workflow of library preparation for SCFM2 samples in sequencing pipelines A and B, highlighting differences. (A) In sequencing pipeline A, rRNA is depleted from total RNA first using an oligo capture and magnetic bead separation approach. Next, adapters are ligated to mRNA fragments and reverse transcription is performed using primers that anneal to adapters, followed by library amplification by PCR. Finally, fragments are size selected by gel electrophoresis and gel extraction. Datasets from sequencing pipeline A were sequenced on Illumina NextSeq 500 or NovaSeq 6000 platforms. (B) In sequencing pipeline B, total RNA is first reverse transcribed using random primers. Next, cDNAs are denatured and renatured to form rRNA/cDNA hybrids, which are enzymatically depleted. Size selection occurs during the cDNA purification process after rRNA depletion. Finally, adapters are ligated to cDNA and library is amplified by PCR. All datasets from sequencing pipeline B were sequenced on the Illumina NovaSeq 6000 platform. This figure was created in Biorender.

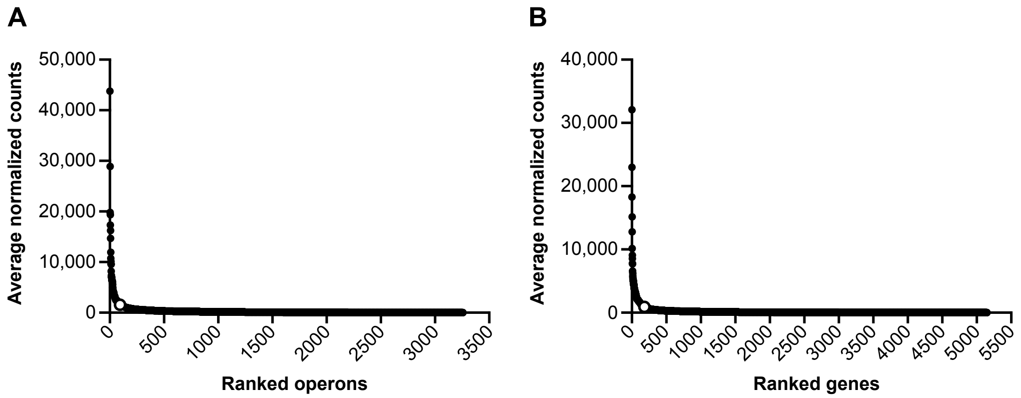

Figure S4. Operon and gene selection for testing if pipeline B selectively depletes abundant transcripts. *P. aeruginosa* (A) operons or (B) genes were ranked by average TMP-normalized expression in SCFM2 samples sequenced with pipeline A and plotted by rank. The R package inflection was used to determine the point of inflection in each curve. Black closed circles represent genes along each curve and the open circles denote the point of inflection for each curve. All operons/genes with expression higher than the operon/gene at the point of inflection were considered "highly expressed" and used to test if sequencing pipeline B depletes abundant transcripts.

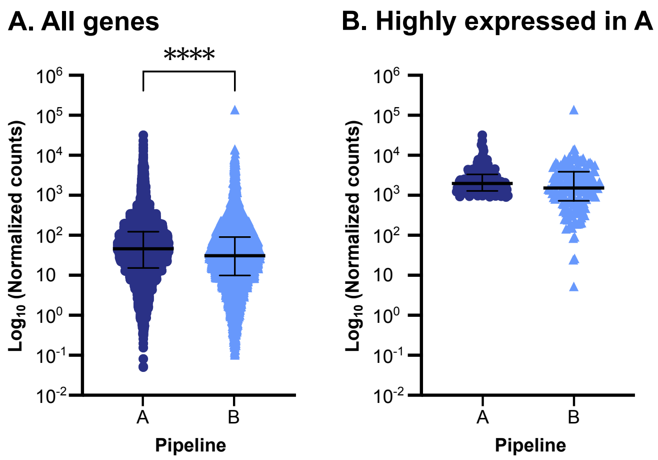

Figure S5. Highly expressed genes have similar expression between pipelines A and B. (A) Average Log_{10} (TMP- normalized expression) of all *P. aeruginosa* genes in sequencing pipelines A or B. Shape and color denotes the pipeline. (B) Average Log_{10}(TMP-normalized expression) in pipelines A and B of *P. aeruginosa* operons highly expressed in pipeline A. Highly expressed operons were determined by ranking each operon's average TPM-normalized expression in pipeline A and calculating the inflection point of the curve (Figure S4B). Median ± interquartile range is shown. Significance was determined using a Mann-Whitney U test. ****: P<0.0001. Significance is not shown for comparison that was not significantly different.

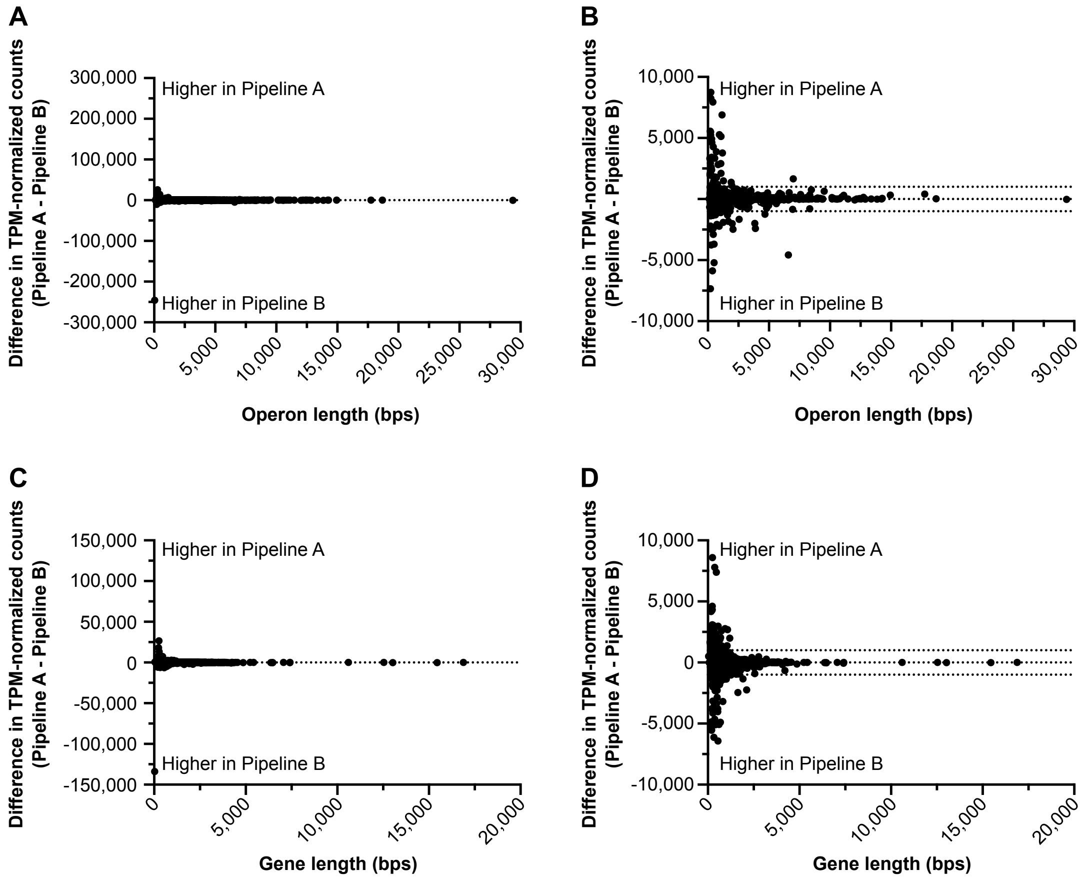

Figure S6. Operon and gene selection for testing if pipeline A selects for more small transcripts than pipeline B. Scatterplots showing difference in TMP-normalized expression of *P. aeruginosa* (A) operons or (C-D) core genes plotted against operon or gene length in base pairs. Operons/genes with an expression difference greater than 1000 (higher expression in pipeline A) or less than -1000 (higher expression in pipeline B) were considered to have “high expression difference between pipelines”, and were used to test if sequencing pipeline A selects for more small transcripts. Dotted lines in (A) and (C): 0; Dotted lines in (B) and (D) from top to bottom: 1000, 0, -1000

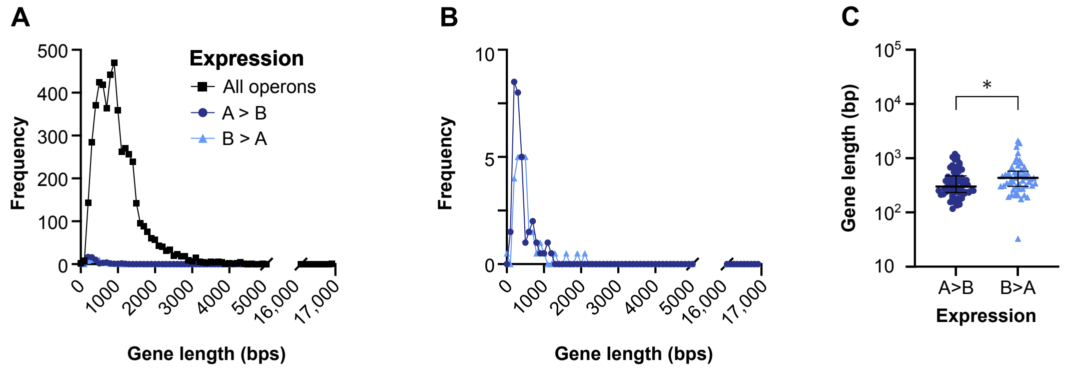

Figure S7. Highly expressed genes in pipeline A are smaller than highly expressed genes in pipeline B. (A) Frequency of genes as a function of length for all genes and genes with the highest expression difference between pipelines A and B (see Figure S6). Skewness of frequency distribution of all genes vs. length is 3.432. (B) Frequency of genes with the highest expression differences between pipelines A and B with y axis adjusted. Skewness of frequency distribution of genes more highly expressed in pipeline A vs. length is 7.116, and skewness of frequency distribution of genes more highly expressed in pipeline B vs. length is 5.805. Color and shape of data points denote the sequencing pipeline where the genes were more highly expressed. Bin widths for gene length in frequency distributions were set to 100. (B) Average lengths of genes with the highest expression difference between pipelines A and B. Median and interquartile range of gene length for each sequencing pipeline is shown. Significance between distributions of operon lengths between pipelines was determined by a Mann-Whitney U test. *: $P \leq 0.05$.

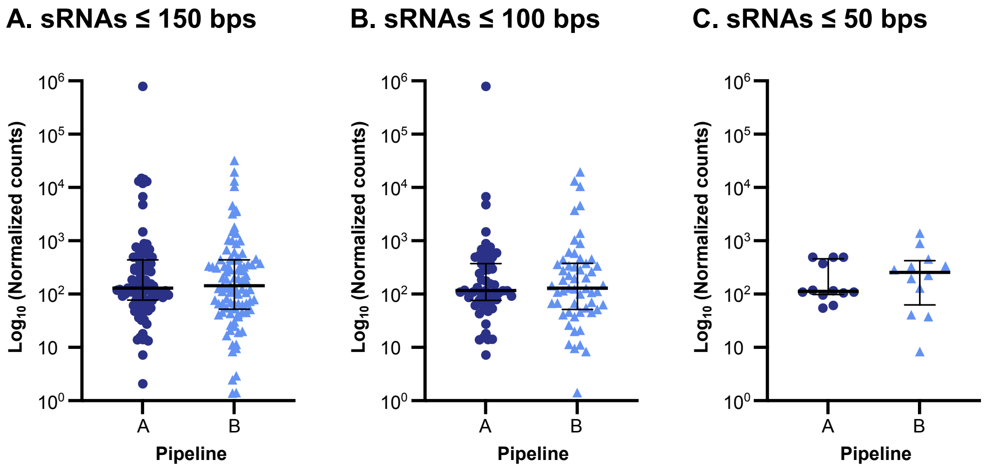

Figure S8. Comparison of sRNAs expression between sequencing pipelines A and B. (A-C) Log transformed average TPM-normalized expression for sRNAs in *P. aeruginosa* PAO1 in sequencing pipelines A and B. Expression is shown for sRNAs 28-150 bps (A), 28-100 bps (B), or 28-50 bps (C). Median with interquartile range is shown for each group. Significance between pipelines was determined by a Wilcoxon match-pairs signed rank test. Comparisons with no significance shown were not significantly different.
